# Miller-Fisher Syndrome: Are Anti-GAD Antibodies Implicated in Its Pathophysiology?

**DOI:** 10.1155/2016/3431849

**Published:** 2016-04-30

**Authors:** Ioannis E. Dagklis, Sotirios Papagiannopoulos, Varvara Theodoridou, Dimitrios Kazis, Ourania Argyropoulou, Sevasti Bostantjopoulou

**Affiliations:** 3rd Department of Neurology, Medical School, Aristotle University of Thessaloniki, 57010 Thessaloniki, Greece

## Abstract

Miller-Fisher syndrome (MFS) is considered as a variant of the Guillain-Barre syndrome (GBS) and its characteristic clinical features are ophthalmoplegia, ataxia, and areflexia. Typically, it is associated with anti-GQ1b antibodies; however, a significant percentage (>10%) of these patients are seronegative. Here, we report a 67-year-old female patient who presented with the typical clinical features of MFS. Workup revealed antibodies against glutamic acid decarboxylase (GAD) in relatively high titers while GQ1b antibodies were negative. Neurological improvement was observed after intravenous gamma globulin and follow-up examinations showed a continuous clinical amelioration with simultaneous decline of anti-GAD levels which finally returned to normal values. This case indicates that anti-GAD antibodies may be associated with a broader clinical spectrum and future studies in GQ1b-seronegative patients could determine ultimately their clinical and pathogenetic significance in this syndrome.

## 1. Introduction

Miller-Fisher syndrome (MFS) is a variant of the Guillain-Barre syndrome (GBS) and its classical clinical triad consists of ophthalmoplegia, ataxia, and areflexia [1]. It is a relatively rare neurological disorder, accounting for approximately 5% of patients with GBS [1]. MFS is an immune-mediated condition and specific anti-ganglioside antibodies, especially IgG anti-GQ1b antibodies, are found in over 80% of the patients [2]. To the remaining patients, some other isolated IgG anti-ganglioside antibodies have been described such as GM1b, GD1a, GD1b, GD1c, GalNAc-GM1b, and GT1a as well as IgG antibodies against ganglioside complexes (GT1a/GM1 and GQ1b/GM1) [3]. However, in a small proportion of GQ1b-seronegative MFS patients no known antibodies are detected. Herein, we present a patient with clinically definite MFS with relatively high serum titers of anti-glutamic acid decarboxylase (GAD) antibodies and no anti-GQ1b antibodies or antibodies against other gangliosides.

## 2. Case Presentation

A 67-year-old woman was admitted to our neurological department with a three-day history of progressive malaise, diplopia, and unsteadiness of gait. She had manifested a flu-like syndrome one week earlier. Her previous medical history included hypothyroidism.

Neurological examination on admission revealed normal cognitive status, paralysis of the oculomotor nerves with dilated unreacting pupils bilaterally, bilateral facial nerve palsy, severe dysmetria of the upper and lower limbs, and truncal and gait ataxia with inability to walk unsupported. A mild distal upper and lower limb weakness was observed bilaterally. The plantar reflex was normal bilateral and deep tendon reflexes were not elicited. Light touch, pain, temperature, and position sense were intact but vibration sense was impaired, mainly in the lower limbs.

The neurophysiological examination revealed normal CMAPs of median, ulnar, tibial, and peroneal nerves (latency and amplitudes) bilaterally. The motor conduction velocities of the above nerves were also within normal limits. The median and ulnar sensory responses were bilaterally low, with normal latencies and conduction velocities. Both radial sensory potentials were low in amplitude. In the lower extremity, the sural and superficial peroneal sensory potentials were unobtainable bilaterally. F-waves were normal in latency and frequency in all examined nerves. The EMG study was entirely normal.

Magnetic resonance imaging of the brain revealed bilateral chronic, periventricular ischemic lesions. Cerebrospinal fluid (CSF) analysis yielded a slight increase in protein level (55 mg/dL), 3 white cells per mm^3^, and normal glucose concentrations. CSF viral serology and gram stain culture were negative. Additional laboratory workup including tests for connective tissue disorders, ACE, serum tumor markers (CEA, AFP, CA19-9, CA15-3, and CA125), anti-TPO and anti-TG antibodies were within normal limits. Serological tests for mycoplasma, CMV, VZV, HSV, EBV, HIV, HCV, and toxoplasmosis were negative as was the stool examination for* Campylobacter jejuni*. Paraneoplastic autoantibodies (anti-Hu, anti-Ri, anti-Yo, anti-Ma2/Ta, anti-amphiphysin, anti-SOX1, anti-recoverin, and anti-CV2) were also negative. Serum antibody test, using Dot-Blot, for GQ1b, GM1, GM2, GM3, GM4, GD1a, GD1b, GD2, GD3, GT1a, GT1b, and sulfatide was negative. A high titer (676 IU/mL, normal range <10 IU/mL) of anti-GAD-65 antibodies, performed by enzyme-linked immunosorbent assay (ELISA, Euroimmun: anti-GAD (IgG), EA 1022-9601 G), was found in serum. Islet cell antibodies (ICA) as well as insulinoma antigen 2 (IA2) antibodies were negative.

From the first day of her admission intravenous immunoglobulin was administered in a dosage of 0.4 g/kg/daily for 5 days. On day 3, the patient developed severe dysphagia and respiratory insufficiency. She was transferred to the intensive care unit for 4 days but there was no need of intubation. The patient then was stabilized and from day 18 she started to improve gradually.

### 2.1. Patient's Follow-Up

Three months later the patient was almost normal. Neurological examination revealed slight limb dysmetria without gait ataxia and decreased tendon reflexes. A revaluation of serum anti-GAD titer was performed and a significant decline was observed (19.5 IU/mL). Nine months later neurological examination was unremarkable, anti-GAD titer was within normal limits, and the neurophysiological study yielded no abnormal results.

## 3. Discussion

MFS is a disease of immunological origin involving an immune-mediated process triggered by an antecedent infection [2]. The association of MFS with IgG autoantibodies against GQ1b ganglioside was first described in the early 1990s. These antibodies are detected between 83 and 100% of the patients, becoming a marker of the disease [3]. GAD is the responsible enzyme for conversion of glutamate to *γ*-aminobutyric acid (GABA), a widely distributed inhibitory neurotransmitter in the brain and spinal cord. The anti-GAD antibodies have been correlated with type 1 diabetes mellitus (T1DM) as well as with a wide range of neurological situations including stiff-person syndrome (SPS), cerebellar ataxia, nonparaneoplastic limbic encephalitis, refractory epilepsy, and abnormal eye movements [4, 5]. Although, in all these diseases, little is known about the pathogenetic role of anti-GAD, it is important to mention that the common presence of a single autoantibody suggests the involvement of different epitopes.

Our case was a typical clinical Miller-Fisher with acute onset of ophthalmoplegia, ataxia, and areflexia without anti-GQ1b antibodies but with relatively high titers of anti-GAD antibodies in the serum. In the literature, only one report exists with seronegative MFS and positive serum anti-GAD antibodies [6]. Moreover, a second case has been reported with acute cranial and peripheral neuropathy and positive serum anti-GAD antibodies without though fulfilling the MFS clinical criteria [7]. Finally, Gupta and Liu described an atypical anti-GQ1b antibody positive case where anti-GAD antibodies were also detected [8]. While the previous reported cases had a slight anti-GAD increase (6-7-fold), our patient showed a remarkable (70-fold) increase. Although, in well-established syndromes associated with anti-GAD, such as SPS, higher titers are usually described ranging from 2000 up to 4–5.000.000 U/mL [5], recently, low titers were found among patients with cerebellar ataxia, a fact that demonstrates the possibility of anti-GAD-associated syndromes in lower levels than have previously been considered acceptable [9]. Furthermore, the titer of anti-GAD was progressively decreasing over time and returned within normal limits in a nine-month period following clinical and electrophysiological improvement. This finding suggests also that this syndrome was immune mediated and the anti-GAD antibodies may be implicated in disease pathogenesis. Additionally, it excludes concomitant T1DM or other autoimmune endocrine or paraneoplastic syndromes, in which anti-GAD could also be positive.

Until now, the direct pathogenic effects of anti-GAD are still a matter of debate although accumulating in vitro and in vivo findings support this notion [10]. On the other hand, several researchers propose that in some cases anti-GAD may be markers of immunological process and other uncharacterized autoantibodies may also contribute to the clinical presentation [11]. Recently, Fouka et al. have not correlated any GAD specific epitope with the expression of a neurological syndrome and assumed that GAD-associated syndromes may involve spread to antigens expressed on the surface of inhibitory neurons and their synapses [12]. Concerning our case, GABAergic synaptic transmission has been shown to be present on the endings of mice spinal nerves [13] and a reduction of GABA synthesis in the nerve terminal as well as dysfunction of GAD-expressing neurons in the brainstem and cerebellum could be proposed as possible mechanisms [11, 14].

In conclusion, this case suggests that anti-GAD antibodies may be associated with MFS, a fact that could provide additional information for its immunopathology and broaden the spectrum of anti-GAD-associated neurological diseases. Further studies evaluating anti-GAD antibodies in GQ1b-seronegative MFS patients may reveal their clinical and pathophysiological role.

## References

[1] Teener J. W. (2012). Miller Fisher's syndrome. *Seminars in Neurology*.

[2] Shahrizaila N., Yuki N. (2013). Bickerstaff brainstem encephalitis and Fisher syndrome: anti-GQ1b antibody syndrome. *Journal of Neurology, Neurosurgery and Psychiatry*.

[3] Koga M., Gilbert M., Takahashi M. (2012). GQ1b-seronegative Fisher syndrome: clinical features and new serological markers. *Journal of Neurology*.

[4] Saiz A., Blanco Y., Sabater L. (2008). Spectrum of neurological syndromes associated with glutamic acid decarboxylase antibodies: diagnostic clues for this association. *Brain*.

[5] Alexopoulos H., Dalakas M. C. (2013). Immunology of stiff person syndrome and other GAD-associated neurological disorders. *Expert Review of Clinical Immunology*.

[6] Pietrini V., Pavesi G., Andreetta F. (2013). Miller Fisher syndrome with positivity of anti-GAD antibodies. *Clinical Neurology and Neurosurgery*.

[7] Saltik S., Türkeş M., Tüzün E., Cakir A., Ulusoy C. (2013). Peripheral neuropathy associated with antiglutamic acid decarboxylase antibodies. *Pediatric Neurology*.

[8] Gupta G., Liu A. (2015). Atypical Miller Fisher syndrome with anisocoria and rapidly fluctuating pupillary diameter. *Case Reports in Neurological Medicine*.

[9] Nanri K., Niwa H., Mitoma H. (2013). Low-titer anti-GAD-antibody-positive cerebellar ataxia. *Cerebellum*.

[10] Mitoma H., Adhikari K., Aeschlimann D. (2016). Consensus paper: neuroimmune mechanisms of cerebellar ataxias. *Cerebellum*.

[11] Chang T., Alexopoulos H., Pettingill P. (2013). Immunization against GAD induces antibody binding to GAD-independent antigens and brainstem GABAergic neuronal loss. *PLoS ONE*.

[12] Fouka P., Alexopoulos H., Akrivou S., Trohatou O., Politis P. K., Dalakas M. C. (2015). GAD65 epitope mapping and search for novel autoantibodies in GAD-associated neurological disorders. *Journal of Neuroimmunology*.

[13] Witschi R., Punnakkal P., Paul J. (2011). Presynaptic *α*2-GABA_A_ receptors in primary afferent depolarization and spinal pain control. *The Journal of Neuroscience*.

[14] Vianello M., Tavolato B., Giometto B. (2002). Glutamic acid decarboxylase autoantibodies and neurological disorders. *Neurological Sciences*.

